# Thymus medulla fosters generation of natural Treg cells, invariant γδ T cells, and invariant NKT cells: What we learn from intrathymic migration

**DOI:** 10.1002/eji.201445108

**Published:** 2015-02-13

**Authors:** Jennifer E Cowan, William E Jenkinson, Graham Anderson

**Affiliations:** MRC Centre for Immune Regulation, Institute for Biomedical Research, Medical School, University of BirminghamBirmingham, UK

**Keywords:** Chemokine, iNKT cells, γδ T cells, Natural Treg cell, Thymic medulla, Thymus

## Abstract

The organization of the thymus into distinct cortical and medullary regions enables it to control the step-wise migration and development of immature T-cell precursors. Such a process provides access to specialized cortical and medullary thymic epithelial cells at defined stages of maturation, ensuring the generation of self-tolerant and MHC-restricted conventional CD4^+^ and CD8^+^ αβ T cells. The migratory cues and stromal cell requirements that regulate the development of conventional αβ T cells have been well studied. However, the thymus also fosters the generation of several immunoregulatory T-cell populations that form key components of both innate and adaptive immune responses. These include Foxp3^+^ natural regulatory T cells, invariant γδ T cells, and CD1d-restricted invariant natural killer T cells (iNKT cells). While less is known about the intrathymic requirements of these nonconventional T cells, recent studies have highlighted the importance of the thymus medulla in their development. Here, we review recent findings on the mechanisms controlling the intrathymic migration of distinct T-cell subsets, and relate this to knowledge of the microenvironmental requirements of these cells.

## Introduction

The immune system consists of a wide range of specialized cells within tissues that play key roles in the control of pathogen recognition, cellular stress, and tumor surveillance. Typically, descriptions of the immune system involve its separation into innate and adaptive components. For example, initial pathogen-mediated activation of innate cell types such as DCs can drive effecter responses by lymphocyte components of the adaptive immune system. Appropriate stimulation of effecter function in αβTCR bearing T cells can take several days, and eventually leads to the formation of antigen-specific memory T-cell populations. However, as shown recently for γδ T cells 1, this separation of innate and adaptive immune cells is not as clearly defined as previously thought, and individual T-cell subsets can be involved in both innate and adaptive arms of the immune response. From a developmental point of view, this is important as the generation of several of the cellular mediators of innate and adaptive immunity takes place at a common site, the thymus. While the role of the thymus in the generation of adaptive CD4^+^ and CD8^+^ αβ T cells is well known (reviewed in 2,3), the thymus also provides support for additional innate and adaptive T-cell subsets including Foxp3^+^ natural regulatory T (Foxp3^+^ nTreg) cells, invariant natural killer T cells (iNKT cells), and various γδ T-cell subsets in fetal and postnatal life 4–6.

Thymic microenvironments demonstrate a distinct anatomical organization that is directly linked to their function 7–9. The medulla represents a site where single positive (SP) thymocytes accumulate prior to their exit into the periphery. Here, various subsets of medullary thymic epithelial cells (mTECs) and DCs are involved in multiple aspects of T-cell development, including negative selection and thymic emigration 10–12. The organization and stromal cell heterogeneity of the thymus medulla is becoming increasingly well-defined 11, including further subdivision of mTECs using markers that include the CCR7 ligand CCL21 13and the atypical chemokine receptor CCRL1 14. These descriptions of medullary heterogeneity are helping to provide a clearer understanding of the mechanisms controlling conventional αβ T-cell development. Increasing evidence suggests that the thymic medulla is also important in the development of other T-cell populations that play important roles in both the innate and adaptive immune systems. In this review, we compare the thymic requirements of these distinct T-cell populations, drawing attention to our current understanding of the multiple roles played by the thymus medulla in the intrathymic migration and development of these cells.

## Conventional CD4^+^ and CD8^+^ αβ T cells

**Figure 1:**
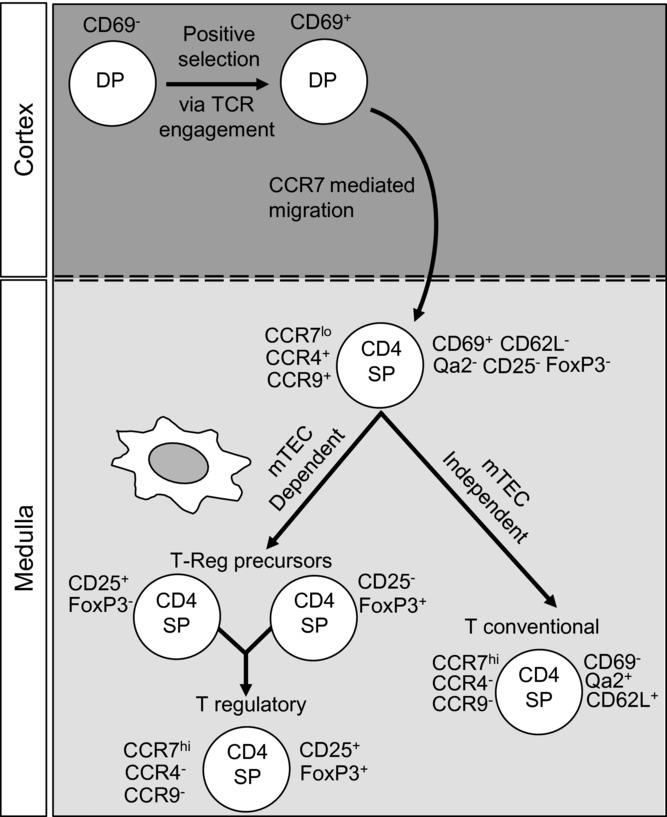
Conventional and Foxp3+ regulatory T-cell development in thymic microenvironments. Both conventional and Foxp3^+^ regulatory CD4^+^ T cells are generated from CD4^+^CD8^+^ double positive (DP) thymocytes following interactions with self-peptide/MHC complexes expressed by thymic stroma. CCR7 expression during positive selection facilitates the migration of positively selected cells from the cortex to the medulla. Further TCR/MHC interactions with mTECs are involved in the generation of Foxp3^+^ nTreg cells from two distinct precursor populations, CD25^+^Foxp3^–^ and CD25^–^Foxp3^+^ cells. In contrast, the generation of phenotypically mature conventional CD4^+^ T cells from recently selected CCR7^lo^CCR9^+^CCR4^+^ thymocytes can occur independently of mTECs.

Following positive selection in the cortex, the medullary dwell time of SP thymocytes has been suggested to be as short as 4–5 days (reviewed in 15), to as long as 2 weeks 16. During this time, medullary resident thymocytes undergo a series of phenotypically and functionally distinct maturation stages (Fig. 1), including downregulation of CD24, and upregulation of Qa2 and CD62L 17. Thus, while newly generated CD4^+^ and CD8^+^ SP thymocytes in mice are characterized by a CD69^+^HSA^high^Qa2^−^CD62L^-^ surface phenotype 15,18,19, with increasing maturity thymocytes downregulate HSA and CD69 whilst upregulating Qa2 and CD62L to become CD69^−^HSA^low^Qa2^+^CD62L^+^ (Fig. 1) 18,20,21. The differentiation program of CD4 SP thymocytes has been further subdivided with the aid of additional markers such as 6C10 22 into four distinct maturation stages, each with unique molecular signatures 23. The possible functional relevance of the majority of these surface markers, however, remains relatively unknown. More recently, the different stages in CD4 SP thymocyte development has been analyzed in relation to the expression of chemokine receptors linked to intrathymic migration (Fig. 1). For instance, newly selected CD69^+^ CD4 SP murine thymocytes have been shown to express high levels of CCR9 and CCR4, while mature CD69^−^ CD4 SP cells are CCR4^−^CCR9^−^[24]. Relevant to the process of medullary entry, visualization of thymocyte mobility within whole thymic lobes via two-photon laser scanning microscopy showed that the positive selection of cells moving randomly within cortical regions triggers their rapid and directed migration toward the medulla 25. Moreover, treatment of mice with pertussis toxin (an inhibitor of G-protein coupled receptors including chemokine receptors) was shown to prevent SP cells from crossing the cortico-medullary junction, resulting in their retention within cortical regions of the thymus 26,27. Of the large family of chemokine receptors, CCR7 has been identified as the prominent receptor mediating this vital relocalization step in intrathymic T-cell development, a finding that maps well with the CCR7 expression pattern in SP thymocytes (Fig. 1) 28,29. TCR engagement during cortical positive selection induces CCR7 surface expression on developing thymocytes 30, which then migrate into medullary thymic microenvironments containing CCL19- and CCL21-producing stromal cells 31,32. Disruption of CCR7-mediated migration results in defective thymocyte migration, cortical accumulation of SP cells 28,29 and disrupted negative selection 30,33, leading to a breakdown of central tolerance 34. Two-photon microscopy monitoring the live migration of CCR7-deficient CD4 SP thymocytes added to thymic slices, suggests that CCR7 may also play a role in the medullary accumulation of thymocytes 27. In addition, further studies suggest that TCR-mediated signaling is impaired in the absence of CCR7, which may contribute to the altered negative selection of thymocytes observed in *Ccr7^−/−^* mice 30.

Further to the induction of CCR7, positive selection of conventional thymocytes alters the expression of other chemokine receptors that may be linked to cortex to medulla migration. For example, CCR9 has been shown to be involved in multiple stages in intrathymic T-cell development in mice, including CD4 SP generation 35. While newly selected CD4 SP thymocytes have been shown to retain CCR9 expression (Fig. 1), their expression of PlexinD1 is thought to suppress CCR9-CCL25 signaling, thereby preventing retention in the cortex and enabling entry into the medulla 36. Along with CCR7 and PlexinD1 orchestrating thymocyte trafficking, other G-protein coupled receptors may also control medullary access, as CCR7 deficiency appears to have a lesser effect on cortex-to-medulla migration compared with the total block observed with pertussis toxin treatment 26–28. CCR4 is upregulated on the thymocyte cell surface after the initiation of positive selection (Fig. 1) 37,38 and its ligands CCL17 and CCL22 are expressed by CD80^high^Aire^+^ mTECs 37,39. However, *Ccr4^−/−^* mice show no obvious disruption in CD4 and CD8 SP T-cell development or their accumulation within medullary regions 38. In addition, thymic stromal cells express CCRL1, an atypical chemokine receptor for CCL19, CCL21, and CCL25 14,40. Interestingly however, and in contrast to earlier reports 41, *Ccrl1*^−/−^ mice show grossly normal thymus architecture and T-cell development 42.

Such findings may reflect redundancy within the extensive chemokine–chemokine receptor system that influences the intrathymic development and location of immature thymocytes 38. Although the importance of thymocyte entry to the medulla in tolerance induction through negative selection is well established, the role of mTECs in the multistage developmental program of differentiating thymoctyes is relatively unclear. To explore this issue, SP thymocyte development has been examined in mice with deficiencies known to disrupt mTEC development, such as *Relb*^−/-^ and *Aire^−/-^* mice 17. Analysis of the latter is of interest and potentially relevant to intrathymic thymocyte migration, as Aire has been linked to the expression of multiple chemokines in the thymic medulla including CCL17, CCL19, CCL21, CCL22, and XCL1 39,43,44. In both *Relb*^−/-^ and *Aire^−/-^* mice, a reduction in mature Qa2^+^CD69^−^ CD4 SP thymocytes has been reported 17, suggesting that the transition from immature to mature stages in conventional CD4 SP thymocyte development is dependent upon the presence of mTEC via a mechanism linked to their expression of Aire. However, the consequences of Relb deficiency are not exclusive to mTECs development/function, and mice deficient in Relb display a complex phenotype including a reduction in thymic DCs, failed lymph node organogenesis and fatal multiorgan autoimmunity 45–47. To specifically address the role of Relb-dependent mTECs in the presence of an otherwise normal immune system, we previously transplanted *Relb^−/−^* fetal thymic stroma into WT mice 24. Analysis showed the presence of mature Qa2^+^ CD69^−^ CD4 SP cells within *Relb^−/−^* mTEC-deficient grafts 24. Moreover, a single cohort of intravenously transferred immature CD4 SP thymocytes was found to undergo late-stage differentiation extrathymically 24. Although these findings suggest that conventional SP thymocyte development can occur independently of interaction with mTECs (Fig. 1), CD11c^+^ DCs present within Relb-dependent mTEC-deficient grafts may influence late-stage thymocyte differentiation 38. More extensive investigations are required to examine the role of thymic DC subsets in fostering conventional CD4 SP thymocyte development, in addition to the division of labor between mTECs and DCs populations in the generation of conventional αβ T cells.

## Foxp3^+^ natural regulatory T cells

While negative selection plays an important role in shaping the developing αβ TCR repertoire, some potentially auto-reactive cells escape intrathymic deletion. Control of unwanted autoimmune responses mediated by these cells requires the thymus to generate a subset of natural CD4^+^ regulatory T (nTreg) cells that possesses potent immunosuppressive properties 48. Commitment to this nTreg lineage occurs in the thymus and depends upon the transcription factor Foxp3, which may be induced as a consequence of increasing affinity of αβ TCR/peptide-MHC interactions (reviewed in 49). Importantly, expression of Foxp3 during nTreg-cell development renders these thymocytes prone to apoptosis unless rescued by signaling through γc-dependent cytokines 50. These findings support a model in which Foxp3^+^ nTreg-cell development in the thymus is a multistage process, involving a sequential requirement for TCR and costimulation followed by cytokine receptor signaling. While the precise timing of the branch point of nTreg lineage commitment from that of conventional αβ T-cell development is uncertain, two distinct nTreg precursor subsets, CD25^+^Foxp3^−^ 51 and CD25^−^Foxp3^+^ thymocytes 50, have been identified within CD4 SP αβ TCR^+^ cells in mice (Fig.1). While identification of the latter is perhaps consistent with studies reporting Foxp3 expression in some CD4^+^CD8^+^ thymocytes 52, the appearance of CD25^+^Foxp3^−^ nTreg precursors in CD4SP thymocytes argues against expression of Foxp3 prior to the SP stage 53. Whatever the developmental sequence may be in Foxp3^+^ nTreg-cell generation, access of thymocytes to the medulla has been shown to play an important role in this process (Fig. 1). For example, both CD25^+^Foxp3^−^ nTreg precursors and CD25^+^Foxp3^+^ nTreg cells are dramatically reduced in the absence of Relb-dependent mTECs 24, and it has been suggested that the importance of mTECs during early stages of nTreg-cell development correlates with their provision of appropriate TCR and costimulatory ligands 54–56. That the availability of mTECs plays a key role in controlling nTreg-cell development is supported by several recent studies. For example, increased mTEC numbers in mice, caused by either loss of TGF-β-mediated or OPG-mediated negative control of medullary growth, was found to correlate with increased numbers of thymic Foxp3^+^ nTreg cells 57,58, while diminished mTEC numbers caused by a NIK mutation in *aly/aly* mice was shown to lead to reduced nTreg-cell development 59. Interestingly, a partial reduction in mTECs observed in mice lacking the adapter molecule Sin did not alter numbers of Foxp3^+^ T cells in lymph nodes 60. Whether this reflects normal Foxp3^+^ T-cell development in the presence of diminished mTEC numbers, or postthymic expansion of nTreg in peripheral tissues is not clear. In addition to the role played by mTECs, several studies have also suggested the involvement of DCs in nTreg-cell development in the thymus. While their role in this process has been unclear, particularly in relation to their known involvement in negative selection, recent evidence demonstrates the importance of the combined presence of both mTECs and DCs, in that Batf3-dependent CD8α^+^ DCs have been shown to support Foxp3^+^ T-cell development via presentation of Aire-dependent antigens provided by mTECs 12.

Although the studies described above highlight the importance of the thymus medulla in nTreg-cell generation, the mechanisms that guide the migration of these cells and their lineage-restricted precursors to this region are not fully understood. For example, while the majority of Foxp3^+^CD4^+^ thymocytes expresses CCR7 (Fig. 1) and demonstrate heterogeneity with regard to expression of CXCR4 and CCR8 61, it is not clear how this heterogeneity relates to the stages in nTreg-cell development described above. More recent analysis shows that CD25^+^Foxp3^−^ precursors in mice express CCR4 and CCR7, while their CD25^+^Foxp3^+^ nTreg progeny are uniformly CCR4^−^CCR7^+^ 24,38. Despite the transient nature of CCR4 expression, Foxp3^+^ nTreg-cell development appears normal in *Ccr4*^−/−^ mice 38. In contrast, *Ccr7*^−/−^ mice do show alterations in the development and intrathymic positioning of nTreg cells 38,52,62, consistent with the idea that conventional αβ T cells and developing nTreg cells share a requirement for CCR7 to enter the medulla. This scenario is also consistent with the diminished number of Foxp3^+^ nTreg cells in mice lacking podoplanin, a molecule that controls the intrathymic localization of CCL21, a ligand for CCR7 that is involved in thymocyte entry to the medulla 63. Interestingly however, nTreg-cell numbers are not decreased in *Ccr7*^−/−^ mice 38. In fact, while the frequency of CD25^+^Foxp3^−^ nTreg precursors is unaltered, CD25^+^Foxp3^+^ nTreg cells are actually increased in *Ccr7*^−/−^ mice 38,62. Of further relevance to the involvement of CCR7 during nTreg-cell development is the recent identification and characterization of the mTEC subset expressing CCL21. Lkhagvasuren et al. 13 showed that the development of CCL21^+^ mTECs is controlled by LTβR signaling, with a decrease in this mTEC subset evident in *Ltbr*^−/−^ mice. Moreover, CCL21^+^ mTECs are largely distinct from the Aire^+^ mTECs subset. However, while *Aire*^−/−^ mice have reported defects in Foxp3^+^ T-cell development 43,64, *Ltbr*^−/−^ mice do not 65,66. Taken together, these findings suggest that in the absence of CCR7 expression by nTreg precursors, or LTβR-mediated CCL21 production by mTECs, nTreg precursors can still develop. This suggests that other chemokines distinct from those triggered by LTβR signaling are able to control the access to mTECs that is required for nTreg-cell generation. The reasons behind the increase in CD25^+^Foxp3^+^ nTreg cells in *Ccr7*^−/−^ mice remain unexplained,. It is unclear whether this occurs as a result of defective emigration leading to increased thymic retention of nTreg cells, or is caused by the reentry of peripheral Foxp3^+^ Treg cells back to the thymus. Furthermore, whether these possibilities also account for the large frequency of Rag2p-GFP^−^ Foxp3^+^ nTreg cells observed in RAG2p-GFP reporter mice 15,67,68 requires additional investigation.

## CD1d-restricted iNKT cells

**Figure 2:**
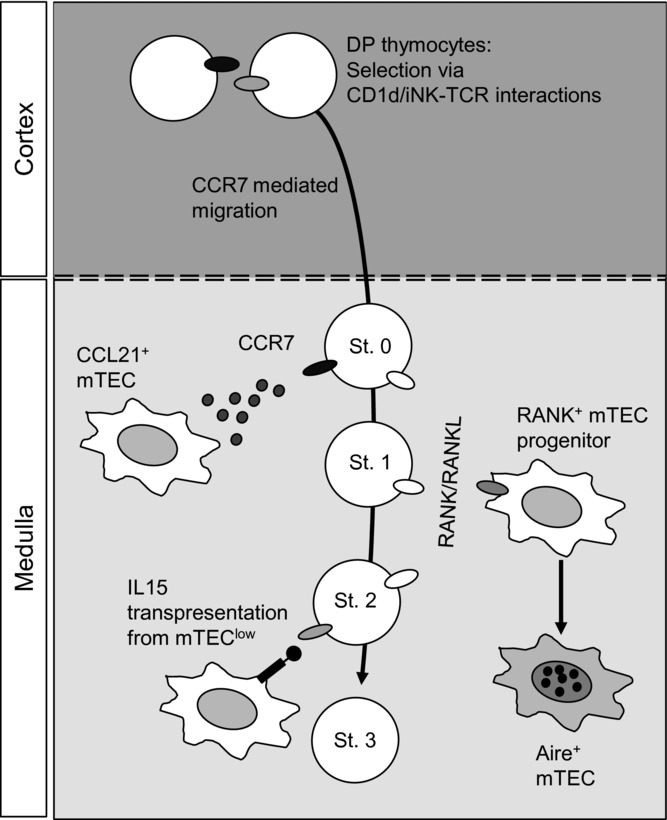
CD1d-restricted iNKT-cell development in thymic microenvironments. Initiation of iNKT-cell development in the thymus occurs in the cortex following recognition of glycolipid/CD1d complexes on CD4^+^CD8^+^ DP thymocytes. The migration of developing iNKT-cells from the cortex to the medulla involves upregulation of CCR7, and so may involve CCL21^+^ mTECs (Stage 0, St. 0). In the medulla, later stages of iNKT-cell development involve IL-15 transpresentation from a subset of mTEC^low^ cells (Stage 2, St. 2). Any relationship between the mTEC^low^ cells which provide CCL21 and those which trans-present IL-15 is not known. In addition to the importance of the thymus medulla for iNKT-cell generation, developing iNKT cells express RANKL to drive Aire^+^ mTEC differentiation. Stages in iNKT-cell development 102: St0: CD24^+^CD44^−^NK1.1^−^, St1: CD24^−^CD44^−^NK1.1^−^, St2: CD44^+^NK1.1^−^, St3: CD44^+^NK1.1^+^.

Of the other αβ T-cell subsets that are generated intrathymically, arguably the most well described are Type 1 invariant natural killer T cells (iNKT cells), which recognize glycolipid antigens bound to CD1d molecules (reviewed in 69–71). Unlike conventional αβ T cells, iNKT cells express an invariant Vα14-Jα18 TCRα chain that pairs with a limited number of TCRβ chains. Moreover, the positive selection of iNKT cells (Fig. 2) depends upon the recognition of CD1d/glycolipid complexes expressed by cortex resident CD4^+^CD8^+^ thymocytes and not TEC 5,72. Although cell surface markers (CD24, CD44, NK1.1), cytokine production (IL-4, IFN-γ, IL-17) and transcription factor expression (PLZF, T-bet, GATA3) identify heterogeneity among iNKT cells during their intrathymic development 73–75, relatively little is known about their positioning in the thymus. This is likely due, at least in part, to the technical difficulty of using CD1d tetramers to detect iNKT cells in tissue sections. However, that positive selection of iNKT cells occurs in the cortex draws parallels with conventional αβ  T cells, and suggests that iNKT cells also undergo cortex-to-medulla migration during their intrathymic development (Fig. 2). Indeed, PCR analysis of thymic iNKT-cell subsets separated on the basis of CD4 and IL17RB expression shows their differential expression of mRNAs encoding the chemokine receptors CCR4, CCR6, and CCR7, the ligands for which are expressed in the medulla 76.

While the relationship between expression of these chemokine receptors and the intrathymic location of particular iNKT-cell subsets is unclear, expression of the chemokine receptor CXCR6 is induced during iNKT-cell positive selection, a process that occurs in the cortex 77. However, while expression of a CXCR6 ligand (CXCL16) has been detectable in thymus medulla 78 no defects were reported during intrathymic iNKT-cell development in CXCR6-deficient mice 77. By performing flow cytometric analysis to compare chemokine receptor expression during conventional αβ T-cell and iNKT-cell development 38, we found that CCR7 expression was most abundant in CD24^+^CD44^−^NK1.1^−^ (stage 0) iNKT cells, suggesting CCR7 induction takes place following their positive selection to mediate trafficking from the cortex to the medulla. Consistent with this, mice lacking CCR7 were shown to have fewer PBS57^+^ iNKT cells, notably the CD44^−^NK1.1^−^ (stage 1), CD44^+^NK1.1^−^ (stage 2) and CD44^+^NK1.1^+^ (stage 3) subsets (Fig. 2). The involvement of the thymus medulla in iNKT-cell development fits well with the requirement for costimulation via B7 family members, which are predominantly expressed in the medulla 79,80. It also correlates with the reduction in iNKT cells in thymus grafts devoid of Relb-dependent mTECs 81–83 that act as a source of IL-15/IL-15Rα trans-presentation 81. While further studies are necessary to fully understand the impact of mTECs on iNKT-cell development, expression of Aire by mTEC has been reported to be dispensable 84. This suggests that although the absence of Aire expression may alter the intrathymic expression of several chemokines 43,44, these changes do not necessarily impact on iNKT-cell development. Finally, the link between medullary microenvironments and intrathymic iNKT-cell development has been strongly highlighted in studies exploring “long-term resident” NK1.1^+^CD44^+^ iNKT cells, which have been shown to remain in the thymus for extended periods 85. While the functional significance of these cells is not yet known, their thymic residency requires the chemokine receptor CXCR3, the ligands for which are expressed by mTECs 86. Collectively, the studies outlined above are important as they support the idea that, as with conventional αβ T cells and Foxp3^+^ Treg cells (Fig. 1), iNKT cell populations in the thymus (Fig. 2) depend upon both the cortical and medullary microenvironments. Furthermore, that iNKT cells undergo stepwise migration through these sites and can influence mTECs via their expression of RANKL and CD40L 81 are additional features shared with conventional αβ T cells and Foxp3^+^ Treg cells.

## γδ T cells

**Figure 3:**
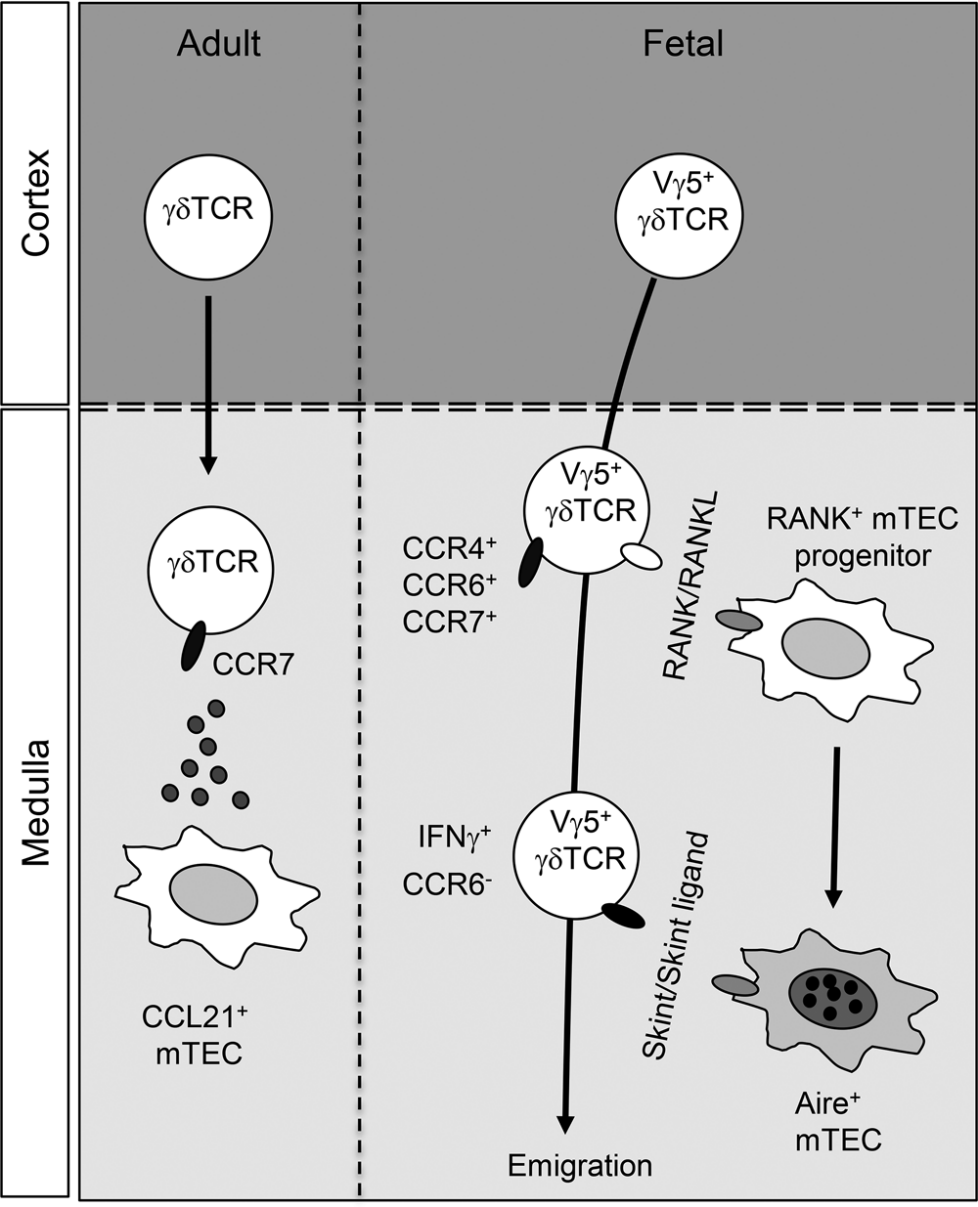
Fetal and adult phases of γδ T-cell development in thymic microenvironments. The thymus supports the generation of distinct subsets of γδ T cells in both fetal and adult life. In the fetal thymus, the first γδ T cells to emerge express the Vγ5^+^ T-cell receptor and represent progenitors of dendritic epidermal T cells. As with the intrathymic development of iNKT cells and conventional T cells, fetal γδ T cells (right) also express RANKL and influence the development of Aire^+^ mTECs in the medulla. Note that although Vγ5^+^ thymocytes express CCR4 and CCR7, they are not required for their intrathymic development. In contrast, CCR6 expression by fetal γδT cells has been linked with their thymic exit. Interestingly, in contrast to fetal thymus (right), CCR7 has been shown to play a role on the medullary accumulation of γδ T cells in the adult thymus (left).

In addition to supporting the development of the αβ T-cell subsets described above, the thymus fosters the development of multiple γδ T-cell subsets. It has long been known that the appearance of heterogeneous γδ T-cell populations follows a temporally ordered program of development, characterized by defined TCR-γ variable gene usage, which is in turn accompanied by biased tissue tropism 77. In this regard, during murine fetal development, γδ T cells utilizing restricted Vγ5 gene segments (Fig. 3) have been shown to arise first, (colonizing epidermal sites and therefore being termed dendritic epidermal T cells DETCs), followed by a wave of Vγ6-bearing γδ T cells (lung, reproductive tract, and tongue), and subsequently Vγ1 and Vγ4 cells (dermis, secondary lymphoid tissues) 87,88. Moreover, such defined γδ T-cell subsets, in a similar fashion to recently described iNKT-cell subsets and novel natural Th17 cells 75,89, appear to preferentially express particular cytokines (IFN-γ, IL-17, and IL-4) and associated transcription factors (Tbet, RORγt, and PLZF) 90. In contrast to development of αβ T cells, the intrathymic developmental requirements of γδ T cells remain comparatively poorly defined, including their dependency upon positive and negative selection. However, evidence has implicated a role for instructive signaling in the direction of γδ T-cell maturation, including conditioning by conventional CD4^+^8^+^ αβ T cells via the lymphotoxin pathway 91, and importantly, interaction of fetal Vγ5^+^ DETCs with RANK-dependent mTECs expressing the immunoglobulin-like molecule Skint-1 (Selection and upKeep of INtraepithelial T cells, Fig. 3) 92,93. Critically, conditioning of Vγ5^+^ DETCs by Skint-1^+^ mTECs appears to bias DETCs toward IFN-γ production, with Skint-1 mutant mice conversely producing IL-17^+^ DETCs 94. Consistent with a potential role for the medulla in the conditioning of fetal γδ T cells, initial experiments analyzing the developmental window during which γδ T cells appeared within the embryonic thymus revealed an accumulation of γδ TCR^+^ cells from approximately E15 of gestation, coinciding with early phases of thymic medullary islet formation 6. Furthermore, histological analysis of the intrathymic anatomical positioning of γδ T cells at later stages of fetal thymus development revealed a preferential accumulation of γδ T cells within medullary foci 95,96. Interestingly however, the positioning of adult γδ T cells has been reported to subsequently demonstrate a reduced bias to medullary regions, with the majority of γδ T cells found to localize to cortical, including subcapsular, and cortico-medullary regions 96,97. Significantly, the functional importance of the medullary accumulation of fetal γδ T cells appears not only important for the conditioning of IFN-γ^+^ Vγ5^+^ DETCs, but in a similar fashion to both conventional and iNKT αβ T cells, fetal γδ T cells were reported to play a role in the reciprocal conditioning of mTECs (Fig. 3), via provision of RANKL to drive the development of the earliest cohorts of Aire^+^ mTECs 92.

Although the requirements for a strictly ordered progression of conventional αβ T-cell sequential migration through cortical and medullary microenvironments has been relatively well characterized, the dependency of γδ T cells upon ordered intrathymic migration, and the mechanisms that control this potential process, both remain much less well-defined. Significantly however, in vitro treatment of E15 fetal thymus organ cultures with pertussis toxin was found to prevent the accumulation γδ T cells within medullary regions, suggesting that in parallel with αβ T cells, the medullary localization of fetal γδ T cells is highly dependent upon G-protein coupled receptor signaling 26,95. Interestingly, initial studies analyzing the development of fetal thymic DETCs revealed that the putative selection of Vγ5^+^ thymocytes resulted in the downregulation of CCR4 and CCR6 with concomitant upregulation of CCR10, indicating changes in chemokine receptor expression may relate to migration of developing γδ T cells through the thymus 98. Despite the expression of the relevant cognate ligands for CCR4 and CCR10 by mTECs 39, the development of Vγ5^+^ thymocytes occurs normally in both *Ccr4*- and *Ccr10*-deficient fetal thymuses 95,99. In contrast, analysis of an intrathymic role for CCR6 expression by Vγ5^+^ DETCs revealed that the selection-associated loss of CCR6 plays a critical role in releasing mature Vγ5^+^ γδ T cells from CCL20-producing medullary microenvironments 100, thereby facilitating thymic egress 101. Consistent with αβ  T-cell populations, γδ T cells additionally express CCR7 at both fetal and adult stages 95,99. Although CCR7-deficient fetal γδ T cells demonstrated no reduction in medullary localization 95, recent studies analyzing the intrathymic positioning of adult γδ T cells revealed a significant reduction in medulla-associated γδ T cells in *Ccr7^−/−^* mice 97. However, beyond the developmental requirement of fetal Vγ5^+^ DETC progenitors for reciprocal signaling interactions with mTECs, the significance of medullary localization and interactions for additional fetal and adult γδT-cell subsets remains unclear and warrants further investigation.

## Conclusions

The medulla has long been recognized as a site that contains thymocyte subsets that represent late stages in T-cell development. Through the process of negative selection, it plays an essential role in conventional αβ T-cell development that ensures the generation of self-tolerant T cells. In addition, increasing evidence suggests that the medulla also influences the development of multiple T-cell lineages that are generated during both fetal and adult life. Recent studies are also now highlighting functional complexity within the mTEC compartment, which may help to explain the contributions made by these cells in supporting the development of diverse T-cell populations. As the cellular and molecular complexity of medullary microenvironments becomes clearer, we anticipate that this will lead to a better understanding of the processes that govern intrathymic T-cell development.

## Abbreviations

mTEC: medullary thymic epithelial cell
SP: single positive
